# Clinical efficacy of ceramic versus resin-based composite endocrowns in Chinese adults: study protocol for a randomized controlled trial

**DOI:** 10.1186/s13063-020-04506-9

**Published:** 2020-06-22

**Authors:** Jilei Wang, Zhiting Ling, Ziting Zheng, Chunqing Zheng, Yawen Gai, Yuting Zeng, Xiaoxia Zhu, Liya Chen, Buling Wu, Wenjuan Yan

**Affiliations:** 1 Department of Conservative and Endodontic Dentistry, Nanfang Hospital, Southern Medical University, 1838 N Guangzhou Road, Guangzhou, 510515 China; 2 The Statistics Room of Nanfang Hospital, Southern Medical University, Guangzhou, China

**Keywords:** Endocrown, CAD/CAM, Ceramic, Resin composite, Endodontically treated teeth, Randomized controlled trial

## Abstract

**Background:**

Endocrown restoration is widely used to restore endodontically treated teeth. However, the clinical effects of different computer-aided design/computer-aided manufacturing (CAD/CAM) materials for endocrown restoration are not clear. The primary objective of this trial is to compare the clinical efficacy of resin-based bloc and ceramic endocrowns for restoring endodontically treated teeth.

**Methods:**

The proposed resin-based bloc and ceramic endocrown assessment trial is a parallel group-designed randomized controlled trial. We will recruit 156 adults between 18 and 75 years old with a minimum of one such molar. The inclusion criteria were good oral hygiene habits, root apex of molar without evident damage, receipt of standard endodontic treatment, need for endocrown restoration, and only one endocrown restoration performed per patient. Patients participating in another study or those with systemic diseases, disabilities, or known allergies to used materials will be excluded. All patients will be randomized and restored with resin-based bloc and ceramic endocrown according to a random number table. Clinical evaluations will be performed at baseline and after treatment at 6, 12, and 24 months, in accordance with the modified Federation Dentaire Internationale (FDI) criteria, by two independent evaluators. The primary outcome is marginal adaptation; secondary outcomes include wear, tooth integrity, fracture of material and retention, marginal staining, and patient view. All data will be analyzed by an independent statistician. Signed rank-sum tests will be used for intragroup comparisons. Wilcoxon rank-sum tests will be used for intergroup comparisons. Hierarchical logistic regression will be used to adjust the baseline and other important indicators.

**Discussion:**

This study will investigate endocrowns of two CAD/CAM materials for endodontically treated molars. The results may help clinicians choose the better CAD/CAM material option and explain to patients the advantages and disadvantages of these two materials with evidence-based support. For patients, the results may lead to improvement in long-term restoration.

**Trial registration:**

ClinicalTrials.gov NCT04033380. Registered on 24 July 2019

## Background

Pulp disease and periapical disease are common oral diseases, and root canal therapy (RCT) is the most effective and ultimate treatment for these diseases [1]. In clinical practice, it is a major challenge to repair the tooth after RCT, since they will be more fragile than vital teeth [2]. If the restoration is not well done in time, the prognosis will decrease greatly, and tooth fracture is one of the most essential causes of treatment failure [3]. After the RCT, excessive removal of surrounding dentin tissue and pulps will cause damage to the overall structure of the tooth, the strength of the tooth will be reduced, and the tooth will lose the nutritional support of the pulp, eventually leading to tooth fracture [4]. Therefore, the endodontically treated teeth should be repaired in time [5]. Endocrowns, full crowns, and post-core crowns are often used to repair these teeth [6]. However, the full crown needs to cut a mass of dental tissues, which results in a significant reduction in the remaining healthy dental tissues [7]. Although post-core crown restoration can strengthen residual dental hard tissue and replace missing dental tissue, which provides the retaining force for the crown, post-core crown restoration can result in additional risks, such as canal perforation and root fracture [8]. Along with the increasing emphasis on minimally invasive trends and the development of adhesive dentistry, an increasing number of clinicians prefer to choose ways to retain more healthy dental tissues [9].

In recent years, the endocrown has been recognized as a new restoration method along with the advantage of minimally invasive treatment [10]. Endocrown is a kind of onlay that is composed of a butt plane and retainer deeply fixed into the internal walls of the pulp chamber [11]. Endocrowns are one-piece constructs that integrate the post, core, and crown to form a complete block prosthesis. In contrast to traditional internal fixation methods, endocrowns are anchored in the inner and margin of the pulp cavity, and the retentive effect better benefits from the macroscopic and microscopic mechanical retentions provided by the pulp cavity and adhesion [12]. Compared with full crown restoration, the endocrown loses less hard tissue, requires less clinical chair time, and the masticatory stress dispersion at the tooth/prosthesis interface is more scientific [13]. Compared with post-core crown techniques, endocrown restoration is simplified because of the core-crown integrity. Furthermore, no post is needed, reducing the risk of root fracture [11]. In a systematic review of three clinical trials, endocrown restorations achieved 94–100% success, which was superior to that of traditional full crown restorations in the anterior teeth [14]. Endocrowns were also shown to restore severely damaged molars. The survival rate was excellent at 99.0% after 44.7 ± 34.6 months, which was superior to that documented in existing data on post- and core-based single crowns [15, 16].

**Fig. 1:**
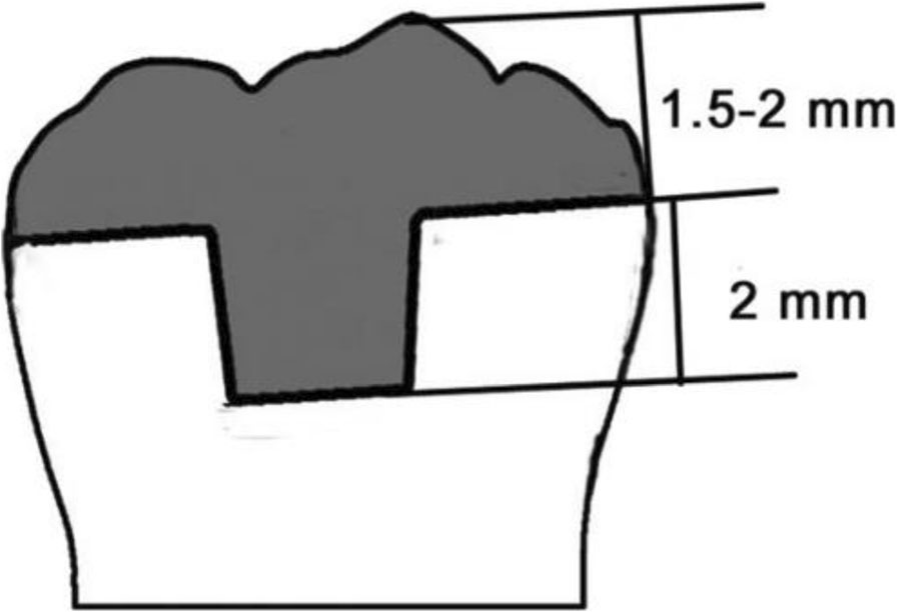
Schematic representation of the flat endocrown

The indications of endocrown include extensive dental defects, inadequate intermaxillary space, lack of the thickness requirements of ceramic materials, inability to use post-core crown or crown for restoration, and cases in which full crown restoration is prohibited due to anatomic variation of the posterior root [17]. We are currently processing a clinical trial on the effect of two marginal endocrown designs, and we found that the flat marginal design was easier to prepare than the 90° shoulder endocrown was [18]. Therefore, the flat marginal form was used in this study (Fig. 1). At present, the fabrication of endocrowns is usually completed by chairside CAD/CAM [19]. CAD/CAM technology has the advantages of high efficiency and accuracy, significantly shortens the production time of restorations [20], and enables clinicians to provide high-quality esthetic restorations in a chairside manner. With the development of CAD/CAM systems, various CAD/CAM materials have been introduced [21]. It has been reported that the restorative effect of endocrowns is closely related to CAD/CAM materials [22]. Leucite-reinforced lithium disilicate and zirconia-reinforced lithium silicate ceramics are thought to be the best choice with the advantages of esthetic and mechanical properties, translucency to natural teeth, good biocompatibility, anti-corrosion, anti-aging, and anti-abrasion [22, 23]. However, ceramic materials have high brittleness, and porcelain fracture frequently occurs and leads to restoration failure [24, 25]. Most ceramic restorations with chairside CAD/CAM require a second sintering. Studies have shown that the crystallization firing process results in a significant increase in the marginal gap size, likely due to shrinkage of the ceramic during the crystallization process [26, 27]. The gap between the tooth and endocrown will reduce the compatibility between the restoration and the tooth and lead to the failure of the restoration.

More recently, ongoing research on biocompatible materials with physical and mechanical properties similar to those of natural tooth tissues has introduced a new generation of nanohybrid composite restorative materials (Grandio blocs, VOCO GmbH, Cuxhaven, Germany). The compositions of resin nanofillers allow the material to have a modulus of elasticity (18.0 GPa) similar to that of dentin. The advantages of resin-based restorations are showing less crack propagation and providing better flexural strength than some CAD/CAM ceramics are [28, 29]. Moreover, resin-based composites are easily cut, cause little damage to grinding burs, and are convenient for repair in the mouth when defects occur.

Because there are no sufficient clinical data to verify that endocrowns fabricated from different CAD/CAM materials are more suitable for the restoration of endodontically treated molars, the main aim of this trial was to compare the clinical efficacy of ceramic versus resin-based bloc endocrowns and to predict the clinical outcomes of these two restorations used for endodontically treated teeth.

### Objective

The main objective is to compare the clinical efficacy of resin-based bloc and ceramic endocrowns in treating endodontically treated molars by assessing the marginal adaptation of restorations fabricated with a chairside CAD/CAM system (Dentsply Sirona, Bensheim, Germany). The minor objectives include evaluating the wear, radiographic examination, patient’s view, and recurrence of caries between the study groups during the same period and looking for the prognostic and influencing factors of the related effects.

### Hypothesis

Our working hypothesis is that the restorative effect of the resin-based bloc endocrown is superior to that of the ceramic endocrown.

## Methods

### Trial design and blindness

This is a randomization, parallel control, optimal design trial with two balanced parallel arms (see SPIRIT checklist, Additional file 1). A double-blinding strategy was designed in this trial. The patients and data analysts are blinded. Operators cannot be blinded because the surface treatment method of ceramic and resin endocrowns is different (in particular, the treatment of the intaglio surfaces of the restoration). It is not possible to blind evaluators because a dentist can easily recognize the material type.

### Block randomization

The method of block randomization is adopted, the block length is set as 6, the number of random seeds is set as “20190811,” and software SAS9.4 was used. All patients who gave consent for participation and who fulfilled the inclusion criteria were randomized. Randomization will be requested by the staff member from the Centre of Clinical Trials (Cen-Trial). After root canal treatment, the allocation of patients will be implemented according to the indication in the random number table. A nurse will call Cen-Trial and tell the doctors about the intervention, and then she will only write the treatment number in the patient’s CRF file. Randomization will be conducted without any influence of the principal investigators, rates, or therapists.

### Participants

The participants will be recruited from the Department of Conservative and Endodontic Dentistry in Nanfang Hospital, Southern Medical University. No special concomitant dental care or intervention is prohibited in participants after inclusion in the trial, except that concerning the included teeth.

### Inclusion criteria

1. The patients are adults aged around 18–75 years and have root apex of molar without evident damage and no root fracture.
2. Good oral hygiene habits.
3. Have a complete root canal therapy molar necessitating an endocrown restoration.
4. The patient has signed an informed consent form.
5. Only one endocrown restoration per patient is eligible.

### Exclusion criteria

1. Allergy to one of the materials used.
2. Poor oral hygiene, bruxism.
3. Severe periodontitis.
4. Pregnancy.
5. Incapable of self-care, mental illness or systemic diseases, and undergoing radiotherapy.
6. Unsuitable for the trial as deemed by the researchers.

### Eligibility criteria

1. The dentist who works in the Division of Endodontics, Department of Stomatology, Nanfang Hospital, Southern Medical University, will perform the interventions.
2. The dentist who will perform the intervention must be licensed as a dentist and had an experience of CAD/CAM technique for at least 3 years.

### Dropout criteria

1. Voluntary withdrawal from the trial by the patient.
2. Poor clinical compliance.

### Outcome measures

Clinical evaluations will be conducted at baseline and after 6, 12, and 24 months of follow-up according to Federation Dentaire International (FDI) criteria by two independent evaluators (Table 1) [30–32]. A standardized training program must be finished by the two evaluators before the trial begins. The two evaluators were trained in the use of the criteria at Nanfang Hospital, SMU. During the first session, the rationale for the FDI criteria, the rating system, the coding system, and the record forms were explained and discussed. Ten restorations were then rated by the instructor to clinically illustrate the rating system. After the instructor explained the reason for assigning each rating, the trainees examined the same restorations. Each trainee was encouraged to explain his or her interpretation of each characteristic and thus his or her reasons for agreement or disagreement with ratings assigned by the instructor. Where disagreements occurred, the categories were again explained so that all examiners would invoke the same concepts when using the rating scales. If the two evaluators present inconsistent evaluations, a third evaluator will perform an evaluation, and the concurring evaluations from the evaluators will be used for analysis. The primary outcome, the marginal adaption of the restoration, will be measured based on the FDI criteria. When a case is evaluated with all items at level A, the restoration is considered a success. When a case has one item at level B and the other at a level no lower than level B, the restoration is considered acceptable, requiring further observation. If a case has any item at level C or D, it is considered a failure. Secondary outcomes which include wear, proximal anatomical form, radiographic examination, patient’s view, recurrence of caries, erosion, abfraction, tooth integrity, periodontal response, adjacent mucosa, oral and general health, surface luster, staining, color match and translucency, esthetic anatomical form, and fracture of material and retention will also be analyzed with FDI criteria.

**Table 1:** Modified FDI criteria

Category	Sub-categories	Grading
**a) Functional properties**	1. Marginal adaptation	A. Clinically excellent B. Clinically good C. Borderline quality/acceptance, repair necessary/possible D. Clinically unsatisfactory (replacement necessary)
	2. Wear	
	3. Proximal anatomical form (contact point/food impact)	
	4. Radiographic examination	
	5. Patient’s view	
**b) Biological properties**	1. Recurrence of caries, erosion, abfraction	
	2. Tooth integrity	
	3. Periodontal response	
	4. Adjacent mucosa	
	5. Oral and general health	
**c) Esthetic properties**	1. Surface luster	
	2. Staining	
	a. Surface	
	b. Margin	
	3. Color match and translucency	
	4. Esthetic anatomical form	
	5. Fracture of material and retention	

The FDI criteria with their various categories and their grading (in italic: the revisions of 2010)

### Primary outcome

Marginal adaptation, FDI standard [2010], which has been defined as follows:

1. Harmonious outline, no gaps, no white or discolored lines (A)
2. Marginal gap (< 150 μm), white lines; small marginal fracture removable by polishing; slight ditching, slight step/flashes, minor irregularities (B)
3. Gap < 250 μm not removable; several small marginal fractures; major irregularities, ditching or flash, steps (C)
4. Gap > 250 μm or dentine/base exposed; severe ditching or marginal fractures; larger irregularities or steps (repair necessary) (D)

### Secondary outcome

Wear:

1. Physiological wear equivalent of enamel (A)
2. Normal wear only slightly different from that to enamel (B)
3. Different wear rate than enamel but within the biological variation (C)
4. Wear considerably exceeds normal enamel, or occlusal contact points are lost (D)

As for the evaluation of the patient’s view, the following questionnaire will be reviewed:

1. Entirely satisfied with esthetics and function (A)
2. Satisfied with esthetics and function with minor roughness (B)
3. Minor criticism but no adverse clinical effects (B)
  - Esthetic shortcomings
  - Some lack of chewing comfort
  - Unpleasant treatment procedure
4. Desire for improvement: esthetics, function, tongue irritation (C)
5. Completely dissatisfied and/or adverse effects, including pain (D)

### Sample size and recruitment procedures

The main evaluation index was the marginal adaptation of the restoration. In this study, the proportions of grades A (clinically excellent), B (clinically good), C (borderline quality/acceptance, repair necessary/possible), and D (clinically unsatisfactory, replacement necessary) in the experimental group are expected to be 60%, 30%, 8%, and 2%, respectively. The proportions of grades A, B, C, and D in the control group are expected to be 40%, 30%, 20%, and 10%, respectively. After a bilateral inspection level of 0.05 was set and the power of the test was set to no lower than 80%, nQuery 8.0 software was applied to calculate the sample size, in which 124 patients (62 for each group) were recruited. Considering that 20% of patients might drop out in the follow-up, the final recruitment sample size of this trial would be 156 patients (78 in each group).

The participants will be recruited from the Department of Conservative and Endodontic Dentistry in Nanfang Hospital, Southern Medical University. There are many patients with root canal treatment in this department each year. Approximately 20–30 endocrown restorations have been fabricated by CAD/CAM in this hospital each month according to the data of last year. Therefore, the achievement of adequate participants is feasible in 2 years. We promise compensation to the patients who participate in the study. Every half a year, all the participants will receive free dental care including teeth cleaning and X-ray examination and oral examination. Every 3 months, a phone call follow-up will be executed to evaluate participants’ general condition. The traffic fee for each visit will be compensated.

### The intervention group and control group

All participants will be randomly allocated into two groups. One group will receive a Vita suprinity endocrown, and the other group will receive with Grandio bloc endocrown (Table 2). Randomization will be performed in accordance with a random list of numbers generated by the Department of Biomedical Statistics of Southern Medical University. The 5 operators are endodontists from the Department of Conservative and Endodontic Dentistry in Nanfang Hospital, Southern Medical University, and all of them will be eligible for inclusion and will receive standardized training in endocrown restoration as previously described before the study begins [18]. The chairside CAD/CAM endocrown will be designed and fabricated by the same technician. The number of cases assigned to each dentist is approximately 30. Another two dentists will serve as evaluators and will be responsible for observing the restorations and collecting data during follow-up.

**Table 2:** SPIRIT (Standard Protocol Items: Recommendations for Interventional Trials)

TIMEPOINT	Pre-treatment	Post-treatment	6-month follow-up	12-month follow-up	24-month follow-up
**ENROLMENT:**					
**Eligibility screen**	**×**				
**Informed consent**	**×**				
**Baseline data collection**	**×**				
**Randomize subjects**	**×**				
**Allocation**	**×**				
**INTERVENTIONS:**					
***Grandio bloc***					
***Vita suprinity***					
**ASSESSMENTS:**					
***Primary outcome***		**×**	**×**	**×**	**×**
***Secondary outcomes***		**×**	**×**	**×**	**×**

### Data collection

The data will be collected from a case report form (CRF) that records all information at baseline and follow-up. The data will be kept anonymous. The CRF includes demographic data, oral habits, medical history, follow-up data, and adverse events. Patients will be identified by the alphabetical order of their full name on the form. The data will be input twice into the database by designated operators and checked by a data manager. The CRF form (hard copy) will be locked in a separated safety box. The database will be submitted and stored in Cen-Trial. To protect the privacy of patients, the patients will be registered with their first letters of their full name at filling the form.

### Statistical methods

#### Basic principles

The nQuery 8.0 statistical software will be used for statistical analysis. The data will be analyzed by an independent statistician. All statistical tests are two-tailed. A *P* value of less than 0.05 will be the level of significance, and 95% confidence intervals will be calculated. Parametric methods will be considered first. Data that do not meet or cannot be transformed to meet parametric assumptions will be analyzed by non-parametric methods.

#### Primary outcome analysis

Signed rank-sum tests will be used for intragroup comparisons, and Wilcoxon rank-sum tests will be used for intergroup comparisons. Hierarchical logistic regression will be used to adjust the baseline and other important indicators.

#### Secondary outcome analysis

For intragroup comparisons, paired *t* tests or signed rank-sum tests will be used for quantitative variables, and McNemar tests will be used for qualitative variables.

For intergroup comparisons, quantitative variables will be analyzed by two-sample *t* tests (two groups) or by non-parametric methods. Qualitative variables will be analyzed by Pearson’s chi-square tests. Rank variables were tested by Wilcoxon rank-sum tests.

### Data monitoring

The DMC consists of the Department of Biomedical Statistics, Southern Medical University, and it is mainly responsible for data management and statistical analysis. It is independent of the sponsor and has no competing interests.

### Harms

In our study, an adverse event will be defined as any untoward medical occurrence in a subject without regard to the possibility of a causal relationship. The adverse events include the materials in the restoration process causing allergic reactions and the prosthesis falling off, leading to aspiration. Any serious adverse events occurring during the course of the test shall be reported to the medical ethics committee of the unit and the applicant immediately, and the “report form of serious adverse events” will be completed. If it is a serious adverse reaction, it will be reported to the state drug supervision and administration within 24 h.

### Auditing

The frequency of audit is once a year. The project organization will review the test process and make a comprehensive evaluation. Eliminate funding for lower ranked projects. The process will be independent from investigators and the sponsor.

## Discussion

In this trial, the FDI criteria will be used to evaluate the quality of restorations. The proposed FDI criteria allow for the classification of the evaluation of dental restorations according to functional, biological, and esthetic categories. Compared to the USPHS criteria, a higher number of scores (1 to 5) were reported to make it easier to discern potential differences in the quality of restorations [33], which increases the overall quality of assessments. Moreover, the FDI criteria can be used to standardize clinical judgment of restorations, allowing for comparisons with all other studies.

We will test two types of CAD/CAM materials that are commonly used in clinical practice. Grandio blocs contain 86% w/w inorganic fillers in a polymer matrix for enhanced strength and excellent wear resistance. With the advantage of dentin-like elasticity modulus, the GR endocrown (composite resin) could achieve a more approximate monoblock structure and dissipate more energy under the same loading, which may have the highest fracture resistance. Vita suprinity (ceramic) has a significant fracture resistance value (1784 N) and more wear resistance than other CAD/CAM ceramic materials have, causing the addition of zirconia to increase its strength [22, 34]. However, the potential for brittle catastrophic fracture and excessive wear on opposing natural teeth are considered the predominant deficiencies [35, 36]. The results of this randomized control trial will allow for advancement in the recommendations and will be beneficial for the patient, the practitioner, and the researcher.

### Trial status

The protocol version number is NFEC-2017-141 and approved on Sep. 7, 2017. This trial is in the process of recruiting participants. The trial recruiting has started on Jul. 26, 2019, and the recruitment will be completed in 2 years.

## Supplementary information

**Additional file 1.** SPIRIT (Standard Protocol Items: Recommendations for Interventional Trials) 2013 Checklist: Recommended items to address in a clinical trial protocol and related documents.

## Data Availability

Any data required to support the protocol can be supplied on request.

## Abbreviations

CAD/CAM: Computer-aided design/computer-aided manufacture
CRF: Case report form
RCT: Root canal treatment
FDI: Federation Dentaire Internationale
