# Supplementary material for: Clinical efficacy of ceramic versus resin-based composite endocrowns in Chinese adults: study protocol for a randomized controlled trial
Source: Trials. 2020 Jun 22;21:559. doi: 10.1186/s13063-020-04506-9 (PMC7310236; doi:10.1186/s13063-020-04506-9)
Supplement: Supplementary file 1 — Additional file 1. SPIRIT (Standard Protocol Items: Recommendations for Interventional Trials) 2013 Checklist: Recommended items to address in a clinical trial protocol and related documents. [file 13063_2020_4506_MOESM1_ESM.docx]

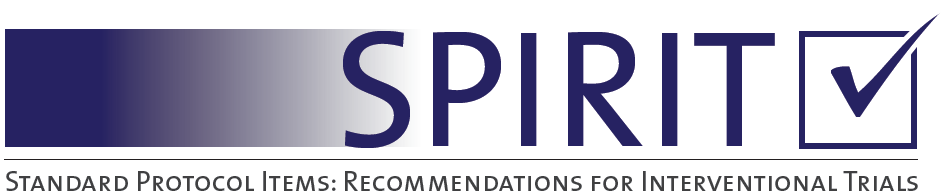


SPIRIT 2013 Checklist: Recommended items to address in a clinical trial protocol and related documents*

| Section/item | Item  No | Description |
| --- | --- | --- |
| **Administrative information** | | |
| Title | 1 | Clinical Efficacy of Ceramic versus Resin-based composite Endocrowns in Chinese Adults: Study Protocol for a Randomized Controlled Trial |
| Trial registration | 2a | Protocol ID: NanFang H; ClinicalTrials.gov ID: NCT04033380; Registered on 24 July 2019; Title: Clinical Efficacy of Ceramic vs Resin Block Endocrowns |
|  | 2b | Please refer to Item 2a and registration in the ClinicalTrials.gov identifier: NCT04033380. Registered on 24 July 2019. -  [https://clinicaltrials.gov/ct2/show/NCT04033380](https://clinicaltrials.gov/ct2/show/NCT04033380) |
| Protocol version | 3 | Version 1.0; Date: 2019-07-24; Author: Wenjuan Yan |
| Funding | 4 | This trial is funded by Clinical Research Startup Program of Southern Medical University by High-level University Construction Funding of Guangdong Provincial Department of Education (LC2016PY023); Clinical Research Program of Nanfang Hospital, Southern Medical University(2018CR018). |
| Author details | 5a | Wenjuan Yan and Buling Wu developed and improved the trial design. Jilei Wang and Zhiting Ling drafted the manuscript, Wenjuan Yan carefully revised and edited the manuscript. Yawen Gai and Yuting Zeng recruited participants. Ziting Zheng and Chunqing Zheng conducted patient follow-up investigation. Xiaoxia Zhu carried out patient care cooperation work, Liya Chen designed the random number table. All authors read and approved the final manuscript. |
|  | 5b | Trial sponsor: Wenjuan Yan, E-mail: 645613053@qq.com |
|  | 5c | This funding source had no role in the design of this study and will not have any role during its execution, analyses, interpretation of the data, or decision to submit results. |
|  | 5d | Data management team from the Department of Biomedical Statistics, Southern Medical University. |
| Introduction |  |  |
| Background and rationale | 6a | Pulpal and periapical diseases are the common oral disease. Root canal treatment (RCT) is the only effective way to treat these diseases. However, failure of RCT is commonly seen without coronal restoration. Endocrown is a new restorative way with a retainer in the pulp cavity which consists of a cervical margin in the form of a butt joint and a preparation of the pulp chamber. This restoration method is not only good at the preservation of residual tooth tissue, but also suitable for severely damaged molars or premolars after dental pulp treatment. More and more endodontists tend to use this ways because they are a minimally invasive solution in such cases. |
|  | 6b | Endocrown is mainly made of glass-ceramics, which has the advantages of beautiful appearance, high hardness and good biocompatibility. However, ceramic materials are brittle and liable to fracture, and need to be sintered twice in the manufacturing process, which is relatively complex and time-consuming. Composite resin block material is a new type of composite material developed in recent years. It is used for chair-side CAD/CAM restoration, composite materials has beautiful and natural colour, and its elastic modulus is close to dentin. Therefore, it is more compatible with natural teeth in performance. Moreover, In the process of making, the steps of secondary sintering and glazing are omitted and shortened. However, there was no sufficient data to verify which material was more effective. |
| Objectives | 7 | Specific objectives: To compare the clinical efficacy of GR and VS endocrowns for restoring endodonticaly treated teeth.  Hypothesis: The restoration effect of the participants with resin-based bloc endocrown is superior to the participants with ceramic endocrown. |
| Trial design | 8 | This trial is a randomized, double blinded, optimal design trial with two balanced parallel arms. |
| **Methods: Participants, interventions, and outcomes** | | |
| Study setting | 9 | Academic hospital: Nanfang Hospital, Southern Medical University, Guangzhou, 510515, China |
| Eligibility criteria | 10 | Inclusion criteria  1. The patients are adults aged around 18-75 years and root apex of molar without evident damage and no root fracture based on:  a) The history of root canal treatment;  b) The symptom of patients: no sinus tract, normal probing patterns;  c) Intraoral dental radiography: no radiolucency involving the apex and mid-root.  2. Good oral hygiene habits evaluated by the following standards:  a) Healthy gums;  b) Brushing more than once daily, use of toothpaste;  c) Annual dental check-ups;  d) A minimal number of missing teeth.  3. Have a complete root canal therapy molar necessitating an endocrown restoration;  4. The patient has signed an informed consent form;  5. Only one endocrown restoration per patient is eligible.  Exclusion criteria  1. Allergy to one of the materials used;  2. Poor oral hygiene, bruxism;  3. Severe periodontitis;  4. Pregnancy;  5. Incapable of self-care, mental illness or systemic diseases and undergoing radiotherapy;  6. Unsuitable for the trial as deemed by the researchers.  Eligibility criteria   1. The dentist who works in the Division of Endodontics, Department of Stomatology, Nanfang Hospital, Southern Medical University will perform the interventions. 2. The dentist who will perform the intervention must be licensed as a dentist and had an experience of CAD/CAM technique for at least three years. |
| Interventions | 11a | Upon fulfilment of selection criteria, 156 eligible participants will receive root canal treatment and then be randomly and equally allocated into the intervention or control group. The patients in the intervention group will be restored by composite resin endocrown. Patients in the control group will be restored by the ceramic endocrown. |
|  | 11b | There will be no special criteria for discontinuing or modifying allocated interventions |
|  | 11c | The participants will only receive one time treatment, so no special strategies will be needed to improve the adherence to intervention in this trial. |
|  | 11d | The treatment of the teeth other than the tooth under investigation will not require alteration to usual care pathways (including use of any medication) and these will continue for both trial arms. |
| Outcomes | 12 | The primary outcome, marginal adaptation of restoration, will be evaluated with FDI criteria. Secondary outcomes including wear, proximal anatomical form, radiographic examination, patient’s view, recurrence of caries, erosion, abfraction, tooth integrity, periodontal response, adjacent mucosa, oral and general health, surface luster, staining, colour match and translucency, esthetic anatomical form, fracture of material and retention will also be analysed with FDI criteria. |
| Participant timeline | 13 | The schedule of enrollment, interventions and assessments is showed in SPIRIT figure 2. |
| Sample size | 14 | The main evaluation index was the marginal adaptation of the restoration. In this study, the proportions of Grades A, B, C and D in the experimental group are expected to be 60%, 30%, 8% and 2%, respectively; the proportions of Grades A, B, C and D in the control group are expected to be 40%, 30%, 20% and 10%, respectively. After a bilateral inspection level of 0.05 was set and the power of the test was set to no lower than 80%, nQuery 8.0 software was applied to estimate the sample size, in which 124 patients (62 for each group) were recruited. Considering that 20% of patients might drop out in the follow-up, the final recruitment sample size of this trial would be 156 patients (78 in each group). |
| Recruitment | 15 | The participants will be recruited from the Department of Conservative and Endodontic Dentistry in Nanfang Hospital, Southern Medical University. There are about 500 patients treated with root canal treatment in this department per month. Approximately 20-30 endocrown restorations have been fabricated by CAD/CAM in this hospital each month according to the data of last year. Therefore, the achievement of adequate participants is feasible in 2 years. We promise compensation to the patients who participate in the study, including additional medical care, free-of-charge oral examination and teeth cleaning. |
| Sequence  generation | 16a | The strategy of block randomization will be adopted, the block length will be 6, the number of random seeds is set as "20190811", and software SAS9.4 was used. The generated random number table will be kept in Cen-Trial and be unavailable to the researchers. |
|  | 16b | After root canal treatment, the allocation of patients will be implemented according to the indication in the random number table. A nurse will call Cen-Trial and tell the doctors about the intervention, and then she will only write the treatment number in the patient’s CRF file. |
|  | 16c | The random number table for allocation will be generated by the staff in the Department of Biomedical Statistics, Southern Medical University. The Staff (Gai Y and Zeng Y) who will take responsibility for recruitment will not take part in any other steps of this trial. The nurse (Zhu X) will contact Cen-Trial to inquire the kind intervention and record the treatment number in CRF form. |
| Assignment of interventions: Blinding | 17a | Double blinding strategy was designed in this trial. The participants and data analysts will be blinded. Operators and evaluator will not be blinded because the material type can be easily told by a professional. |
|  | 17b | No anticipated unblinding issue will occur as no severe side effects associated both interventions have been reported. |
| Data collection and management | 18a | Clinical evaluations will be performed at baseline, 6, 12 and 24 months post restoration. 2 evaluators will perform evaluations according to the FDI criteria independently. The evaluators will receive a standard training before the start of the trial. If two evaluators have significantly different opinions during study, a third evaluator will be involved and the similar opinions by two of three evaluators will be accepted.  The investigators will use a Case Report Form (CRF) to collect the data for outcomes analysis. The CRF covered demographic data, oral habits, history and adverse events. To protect the privacy of patients, the patients will be registered with their first letters of their full name at filling the form. |
|  | 18b | Every half a year, all the participants will receive free dental care including teeth cleaning and X ray examination and oral examination. Every three months, a phone call follow-up will be executed to evaluate participants’ general condition. The traffic fee for each visit will be reimbursed. |
| Data management | 19 | The baseline data, follow-up trial data, and adverse events will be recorded and input twice into the database by designated operators and checked by a data manager. Data will be kept anonymous. The CRF form (hard copy) will be locked in a separated safety box. The database will be submitted and stored in Cen-Trial. |
| Statistical methods | 20a | nQuery 8.0 statistical software was used for statistical analysis. The data will be analyzed by an independent statistician. All statistical tests are two-tailed. A *P* value of less than 0.05 will be the level of significance, and 95% confidence intervals will be calculated. Parametric methods will be considered first. Data that do not meet or cannot be transformed to meet parametric assumptions will be analyzed by non-parametric methods. |
|  | 20b | **Primary outcome analysis**  Signed rank-sum tests will be used for intragroup comparisons, and Wilcoxon rank-sum tests will be used for intergroup comparisons. Hierarchical logistic regression will be used to adjust the baseline and other important indicators.  **Secondary outcome analysis**  For intragroup comparisons, paired *t*-tests or signed rank-sum tests will be used for quantitative variables, and McNemar tests will be used for qualitative variables.  For intergroup comparisons, quantitative variables will be analyzed by two-sample *t-*tests (two groups) or by nonparametric methods. Qualitative variables will be analyzed by Pearson’s chi-square tests. Rank variables were tested by Wilcoxon rank-sum tests. |
|  | 20c | A multiple imputation method will be applied to deal with the missing data. The bad adherence to the once in all intervention will be unlikely. |
| Data monitoring | 21a | DMC consists of the Department of Biomedical Statistics, Southern Medical University, and it will be responsible for data management and statistical analysis. It is independent from the sponsor and competing interests. |
|  | 21b | There are no anticipated problems that are detrimental to the participant. |
| Harms | 22 | In our study an adverse event will be defined as any untoward medical occurrence in a subject without regard to the possibility of a causal relationship. The adverse events include the materials in the restoration process are allergic, and the prosthesis falls off, leading to aspiration. Any serious adverse events occurring in the course of the test shall be reported to the medical ethics committee of the unit and the applicant immediately, and the "report form of serious adverse events" shall be filled in. If it is a serious adverse reaction, it shall also be reported to the state drug supervision and administration within 24 hours. |
| Auditing | 23 | The frequency of audit is once a year. The project organization will review the test process and make comprehensive evaluation. Eliminate funding for lower ranked projects. The process will be independent from investigators and the sponsor. |
| Research ethics approval | 24 | The trial has been approved by the Medical Ethics Committee of Nanfang Hospital, Southern Medical University. This protocol has been reviewed and approved by the sponsor and the ethical committees. Subsequent to initial review and approval, the sponsor and the ethical committees will review the protocol at least annually. The Investigator will make safety and progress reports to the ethical committees at least annually and within three months of study termination or completion at her site. |
|  | 25 | Any amendments to the protocol will be reviewed and approved by the ethics committee and funding support departments. |
| Consent or assent | 26a | Trained Researchers will introduce the trial to patients. Patients will also receive information sheets. Researchers will discuss the trial with patients. Researchers will obtain written consent from patients willing to participate in the trial. |
|  | 26b | On the consent form, participants will be asked if they agree to use of their data should they choose to withdraw from the trial. Participants will also be asked for permission for the research team to share relevant data with people from the Universities taking part in the research or from regulatory authorities, where relevant. This trial does not involve collecting biological specimens for storage. |
| Confidentiality | 27 | All study-related information will be stored securely in the computer. All participants’ information will be stored in locked file cabinets. All reports, data collection, process, and administrative forms will be identified by a coded ID number. |
| Declaration of interests | 28 | All academic conferences and research activities related to this study will be reimbursed. No other competing interests. |
| Access to data | 29 | The Data Management Coordinating Center will oversee the intra-study data sharing process, with input from the Data Management Subcommittee. All Principal Investigators will be given access to the data. All data sets will be password protected. Project Principal Investigators will have direct access to their own site’s data sets, and will have access to other sites data by request. To ensure confidentiality, data dispersed to project team members will be blinded of any identifying participant information. Any data required to support the protocol can be supplied on request." |
| Ancillary and post-trial care | 30 | Patients who participated in the study could receive compensation from the study unit, including additional medical care, compensation or damages. |
| Dissemination plans | 31a | Results will be communicated to relevant groups via conferences, publications, reporting results in databases, data sharing arrangements, wechat - social media or through the sponsor |
| Authors’ contributions | 31b | All named authors adhere to the authorship guidelines of Trials. All authors have agreed to publication. |
|  | 31c | The datasets analysed during the current study are available from the corresponding author on reasonable request. |
| Consent for publication | 32 | This is available from the corresponding author on request. |
| Biological specimens | 33 | There were no plans for collection, laboratory evaluation, and storage of biological specimens for genetic or molecular analysis in the current trial and for future use in ancillary studies. |

*It is strongly recommended that this checklist be read in conjunction with the SPIRIT 2013 Explanation & Elaboration for important clarification on the items. Amendments to the protocol should be tracked and dated. The SPIRIT checklist is copyrighted by the SPIRIT Group under the Creative Commons “[Attribution-NonCommercial-NoDerivs 3.0 U](http://www.creativecommons.org/licenses/by-nc-nd/3.0/)[nported](http://www.creativecommons.org/licenses/by-nc-nd/3.0/)” license.
